# Pregnancy level of estradiol attenuated virus-specific humoral immune response in H5N1-infected female mice despite inducing anti-inflammatory protection

**DOI:** 10.1080/22221751.2019.1648184

**Published:** 2019-07-31

**Authors:** Courtney L. Finch, Anding Zhang, Martina Kosikova, Toshiaki Kawano, Marcela F. Pasetti, Zhiping Ye, Jill R. Ascher, Hang Xie

**Affiliations:** a Division of Viral Products, Center for Biologics Evaluation and Research, United States Food and Drug Administration, Silver Spring, MD, USA; b Department of Pediatrics, Center for Vaccine Development and Global Health, University of Maryland School of Medicine, Baltimore, MD, USA; c Division of Veterinary Services, Center for Biologics Evaluation and Research, United States Food and Drug Administration, Silver Spring, MD, USA

**Keywords:** Pregnancy level of estradiol, avian influenza, humoral immunity, Th2 response, sex-biased

## Abstract

Estradiol, a major female steroid produced during pregnancy, has been reported to protect ovariectomized animals against H1N1 influenza infections via its anti-inflammatory effects. However, it remains unclear why pregnant women with high gestational estradiol levels are highly susceptible to influenza infections. This study was aimed to investigate the effects of pregnancy level of estradiol on female immunity against H5N1 infection in Balb/c mice. A sex-dependent susceptibility to H5N1 infection (higher morbidity and higher mortality) was observed in both pregnant and non-pregnant female mice as compared to male mice. Subcutaneous implantation of estradiol pellets increased serum estradiol concentrations of non-pregnant female mice to the pregnancy level. These mice were protected from H5N1 infection through downregulation of pulmonary pro-inflammatory cytokines. However, the production of virus-specific antibodies after infection was significantly delayed in estradiol-implanted mice when compared to placebos. Virus-specific IgG-secreting and IL-4-secreting cells were also reduced in estradiol-implanted mice. Similarly, lower antibody titers to seasonal vaccine antigens were found in pregnant women as compared to non-pregnant females without hormone usage. Our results indicate that estradiol levels equivalent to those found during pregnancy have divergent effects on female immunity against influenza, highlighting the importance of vaccination during pregnancy to prevent severe influenza infections.

## Introduction

Influenza viruses cause 3–5 million cases of infections and approximately 650,000 associated deaths worldwide every year [1]. Women, particularly pregnant women, are susceptible to influenza infections and have been reported to be disproportionately more likely to experience severe influenza-like illness and deaths than men, especially during pandemics [2–5]. For instance, the overall mortality rate of the 1918 “Spanish Flu” was 2.5%, but 27% of all deaths occurred in pregnant women [3,5,6]. Similarly, during the 2009 H1N1 pandemic, 5% of all deaths occurred in pregnant women, yet pregnant women accounted for only 1% of all cases [5,6]. From November 2003 to May 2008, 383 laboratory-confirmed H5N1 infections had been reported by the WHO, 51% of which occurred in females with a mean age of 22.1 years [7]. Compared to adult males, females of reproductive age including pregnant women were more likely to die from H5N1 infections [8–10].

Sex steroids such as testosterone, estrogen (estrone, estradiol and estriol) and progesterone are known to not only determine sexual dimorphism but also modulate immune responses [3,11–15]. In females, estradiol and progesterone fluctuate over the course of the menstrual cycle. Following the uterine implantation of an embryo, the corpus luteum in the ovary begins to produce more estrogen and progesterone. The levels of estrogen and progesterone continue to rise and peak in the second and third trimesters when the placenta takes over the secretion. Unlike progesterone, which is generally immunosuppressive [16], estradiol has multifaceted, dose-dependent immunomodulatory functions [17]. At normal physiological levels, estradiol is immunostimulatory and augments B cell function and survival [18]. Thus, women tend to mount higher antibody responses than men after vaccinations [19,20]. After conception, estradiol levels rise steadily when pregnancy progresses, which suppresses Th1-biased pro-inflammatory responses resulting in Th2-polarized shift of maternal immunity that promotes immune tolerance and prevents fetal rejection [11,17,21].

Elevated estradiol has been reported to have anti-inflammatory protection against H1N1 influenza infections in animal models [15,22–24]. This notion apparently contradicts the clinical observations of female-biased vulnerability to influenza infections, especially in pregnant women [2–5,8–10,22,25–27]. The protective effect of elevated estradiol has been demonstrated mainly in ovariectomized animal models [15,23,24,28], and the ovary is one of the major female reproductive organs to produce estradiol and other essential female hormones. Additionally, the elevated estradiol concentrations in those ovariectomized animals were considerably lower than those present during pregnancy [15,17,23,24,28]. These differences may have contributed to the disparate findings between ovariectomized animal models and human clinical observations.

In this study, we used an estradiol-implanted mouse model with intact ovaries to investigate how pregnancy level of estradiol alone, independent of other pregnancy-associated hormones, affects host immune responses to H5N1 infection. A human H5N1 A/Vietnam/1203/2004 vaccine reassortant fully virulent in mice [29] was used as a representative H5N1 virus for experimental infection in this study because this H5N1 vaccine strain is no longer a select agent and can be manipulated at Animal Biosafety Level (ABSL)-2 level. Our results indicate that the gestational level of estradiol could protect female mice from H5N1 infection by mitigating inflammation, while it also delayed virus-specific humoral immunity after infection.

## Materials and methods

### Viruses

All viruses including human H5N1 A/Vietnam/1203/2004 (VN/1203) vaccine reassortant bearing a monobasic cleavage site in HA [29], H1N1 A/Michigan/45/2015 and H3N2 A/Hong Kong/4801/2014 were propagated in 9–11-day-old embryonated eggs at 33°C. Infectious viral particles were determined by a plaque assay [29,30].

### Mice

Age-matched non-pregnant female and male adult Balb/c mice, or timed-pregnant Balb/c mice (after 13 days of gestation) were purchased from Charles River laboratories (Frederick, MD) and were infected at approximately 16-week old. Non-pregnant female mice were implanted subcutaneously with 21-day slow release 17-β-estradiol pellets (35 mg/pellet/mouse) or similar-sized placebo (Innovative Research of America, Sarasota, FL) [22] one week before infection. All mice were infected intranasally with H5N1 VN/1203 at 500 PFU/50 µl/mouse (a dose pre-determined to be sublethal in male adult mice). Body weight (BW) was monitored daily for up to 14 days post infection (p.i.). Mice reaching humane endpoints (e.g. 30% BW loss) were promptly euthanized. All procedures were performed under protocols approved by the FDA White Oak Animal Program Animal Care and Use Committee. All animal experiments were repeated 2–3 times. Representative results of multiple experiments were reported. See additional information in Supplementary Materials online.

### Antibody determination

Sera were pre-treated with receptor-destroying enzyme (Denka-Seiken, Tokyo, Japan) and were subjected to hemagglutinin (HA) inhibition (HAI) assays as described [29,31]. Turkey erythrocytes (0.5%), guinea pig erythrocytes (0.75%) and horse erythrocytes (1%) were used in HAI assays for influenza H1N1, H3N2 and H5N1 viruses respectively [29,31]. HA-specific IgG ELISA was performed in 96-well plates pre-coated with 0.5 µg/ml of H5 recombinant HA (rHA) (Protein Sciences, Meriden, CT), or with 0.2 µg/ml of H1 or H3 rHA (Immune Technology, New York, NY) [32]. Bound antibodies were detected with peroxidase-conjugated secondary antibodies (Life technologies) and optical density (OD) at 450 nm was measured using a Victor V multilabel reader (PerkinElmer, Waltham, MA).

### Serum estradiol determination

Mouse serum estradiol levels were determined using an EIA kit (Cayman Chemical, Ann Arbor, Michigan) according to the manufacturer’s instructions.

### Cytokine detection

Proinflammatory cytokines and chemokines in mouse sera and lung homogenates were detected using MSD multiplex kits (Meso Scale Diagnostic, Rockville, MD) according to the manufacturer’s protocols. IFN-β in mouse sera and lung homogenates was quantitated using VeriKine-high sensitivity serum ELISA kits (PBL Assay Science, Piscataway, NJ).

### ELISPOT

Mouse IFN-ɣ, IL-4 or IgG ELISPOTs were performed using Mabtech’s ELISPOT BASIC kits (Cincinnati, OH). Briefly, the 96-well Multiscreen plates (EMD Millipore, Billerica, Massachusetts) were pre-coated with 15 µg/ml of mouse-specific anti-IFN-ɣ, anti-IL-4 or anti-IgG antibody, or with 15 µg/ml of H5 rHA. Spleens harvested from H5N1-infected placebo or estradiol-implanted female mice were dissociated and lysed of red blood cells. After washing, resuspended splenocytes were added to anti-mouse IFN-ɣ or IL-4 antibody pre-coated plates at 2.5 × 10^5^ cells/100 µl/well and were incubated with PMA/ionomycin (PMA/IM,1 µg/ml PMA plus 0.75 µg/ml IM) or H5 rHA (10 µg/ml) at 37°C for 40 h. Cells incubated with medium only served as negative controls. For mouse IgG ELISPOT, splenocytes were pre-activated with R848 (1 µg/ml) and mIL-2 (10 ng/ml) before being added to plates coated with anti-mouse IgG or H5 rHA. Spot-forming cells were detected using biotinylated secondary antibodies and counted using the Immunospot Analyzer equipped with Biospot Version 5.0 software (Cellular Technology Ltd, Cleveland, OH). The number of antigen-specific spots were determined by subtracting the number of spots in unstimulated negative control wells and were expressed as number of spots per 10^6^ cells. Each mouse sample was assayed in triplicates.

### Cell proliferation

Splenocyte proliferation was assessed using a Bromodeoxyuridine (BrdU)-based Chemiluminescent ELISA kit (Roche, Indianapolis, Indiana). Briefly, dissociated splenocytes were resuspended in RPMI 1640 complete media containing 20 U/ml of mouse recombinant IL-2 and were seeded at 1 × 10^4^ cells/100 µl/well in black 96-well ViewPlates (Perkin Elmer, Waltham, Massachusetts) in the presence of PMA/IM mixture (1 µg/ml PMA plus 0.75 µg/ml IM) or H5 rHA (5 µg/ml). BrdU labeling solution was added at 90 h later. Following cell fixation, incorporated BrdU was detected using peroxidase-conjugated anti-BrdU monoclonal antibody according to the manufacturer’s instructions. Cell proliferation was reported as fold induction compared with luminescence intensity of unstimulated control cells.

### Quantitative RT-PCR

Total lung RNA was extracted from placebo or estradiol-implanted mice using the RNeasy microarray tissue mini kit (QIAgen, Germantown, MD). A total of 100 µg high-quality RNA was reverse transcribed using RT^2^ Easy First Strand Kit (QIAgen). Resultant cDNA was then used as the template along with Profiler™ PCR Array Mouse Signal Transduction PathwayFinder™ kit (QIAgen) to perform real-time PCR in Stratagene MX3000p qPCR system with the following conditions: hold for 10 min at 95°C, followed by 40 cycles of 15 s at 95°C and 60 s at 60°C. Individual gene expressions based on threshold cycle (C_T_) values were normalized to the average of five internal housekeeping genes (Actb, B2 m, Gapdh, Gusb and Hsp90ab1) and were calculated for fold changes followed by scatter plot generation using Qiagen’s RT^2^ Profiler PCR Array Data Analysis Webportal according to instructions.

### Human sera

Sera from eight pregnant (mean age = 29.6 ± 1.5 years) and seven non-pregnant women without any type of hormone-based birth control (mean age = 32.7 ± 2.2 years) were collected approximately eight months after 2017/18 Northern Hemisphere seasonal influenza vaccination. Archived anonymized sera were analyzed for H1N1 vaccine prototype virus – A/Michigan/45/2015 and H3N2 vaccine prototype virus – A/Hong Kong/4801/2014 specific total IgG and HAI titers as described above. Specimens were obtained under protocol HP-00040025 approved by the University of Maryland Institutional Review Board.

### Statistical analysis

Statistical analysis with *p* value calculation was performed using unpaired Student *t*-test with Welch’s correction, two-way ANOVA or Mann–Whitney test as specified in individual figure legends (Prism 6.0, GraphPad, San Diego, CA).

## Results

### Sex-dependent differences in mice infected with H5N1 virus

**Figure 1:**
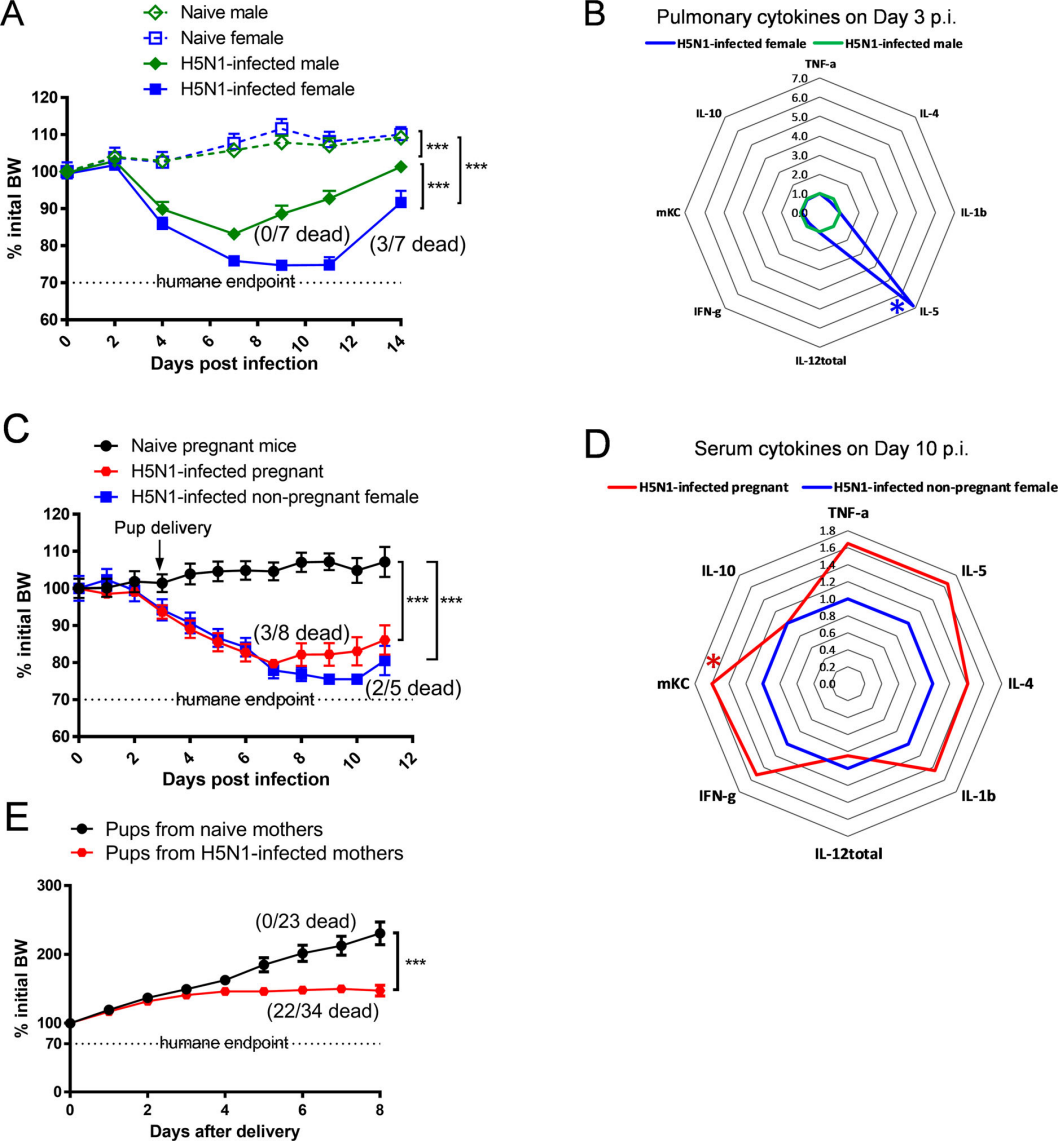
. Sex-dependent differences in response to H5N1 infection. (A) Body Weight (BW) (*n *= 5–7 mice/group) and (B) radar chart of pulmonary cytokines on Day 3 post infection (p.i.) (*n *= 6 mice/cytokine/group) of age-matched male and female Balb/c mice. (C) BW (*n* = 5–8 mice/group) and (D) radar chart of serum cytokines on Day 10 p.i. (*n *= 5–8 mice/cytokine/group) of pregnant and age-matched non-pregnant female Balb/c mice. (E) BW of pups born to naïve and H5N1-infected dams (*n* = 23–34 pups/group). BW data are expressed as mean ± SEM. Individual cytokines are shown as fold inductions vs. male mice (B) or vs. non-pregnant female mice (D). BW, pulmonary cytokines and serum cytokines were analyzed by two-way ANOVA and unpaired Student’s *t*-test, respectively. *p < 0.05 and ***p < 0.001. Data are representative of 2–3 independent experiments with similar results.

Following the sublethal H5N1 infection, both male and female mice showed significant morbidity and lost approximately 16% and 26% initial BW, respectively (*p* < 0.001, Figure 1(A)). While age-matched male mice quickly recovered from H5N1 infection and exhibited 0% mortality, H5N1-infected female mice showed a delayed recovery and experienced 42.8% mortality (Figure 1(A)). H5N1 infection induced strong pro-inflammatory cytokine secretion in the lungs of infected male and female mice 3 days p.i. (the peak pulmonary virus replication): female mice had a 6-fold higher pulmonary IL-5 production (*p* < 0.05 vs. male mice) whereas no substantial differences were seen in the other pro-inflammatory cytokines tested (Figure 1(B) and Supplementary Figure S1). Like age-matched non-pregnant female mice, pregnant dams infected with H5N1 on gestational day 18 also lost >20% of initial BW and exhibited 37.5% mortality after infection (Figure 1(C)). Pregnant dams had serum cytokines measured on day 10 p.i. (7 days after delivery) to ascertain duration of systemic inflammatory responses beyond viral clearance. Pregnant dams that survived H5N1 infection retained higher serum levels of pro-inflammatory cytokines on day 10 p.i., including mKC remaining significantly higher than those of surviving non-pregnant female mice (*p* < 0.05, Figure 1(D) and Supplementary Figure S2). Pups born to H5N1-infected dams not only experienced retarded growth but also had 64.7% mortality (Figure 1(E)). These results confirm a sex-dependent difference in mouse response to H5N1 infection, with female mice (including pregnant) being more susceptible to H5N1 infection than male mice, consistent with human clinical observations [7–10].

### Pregnancy level of estradiol protected against H5N1 infection

**Figure 2.**
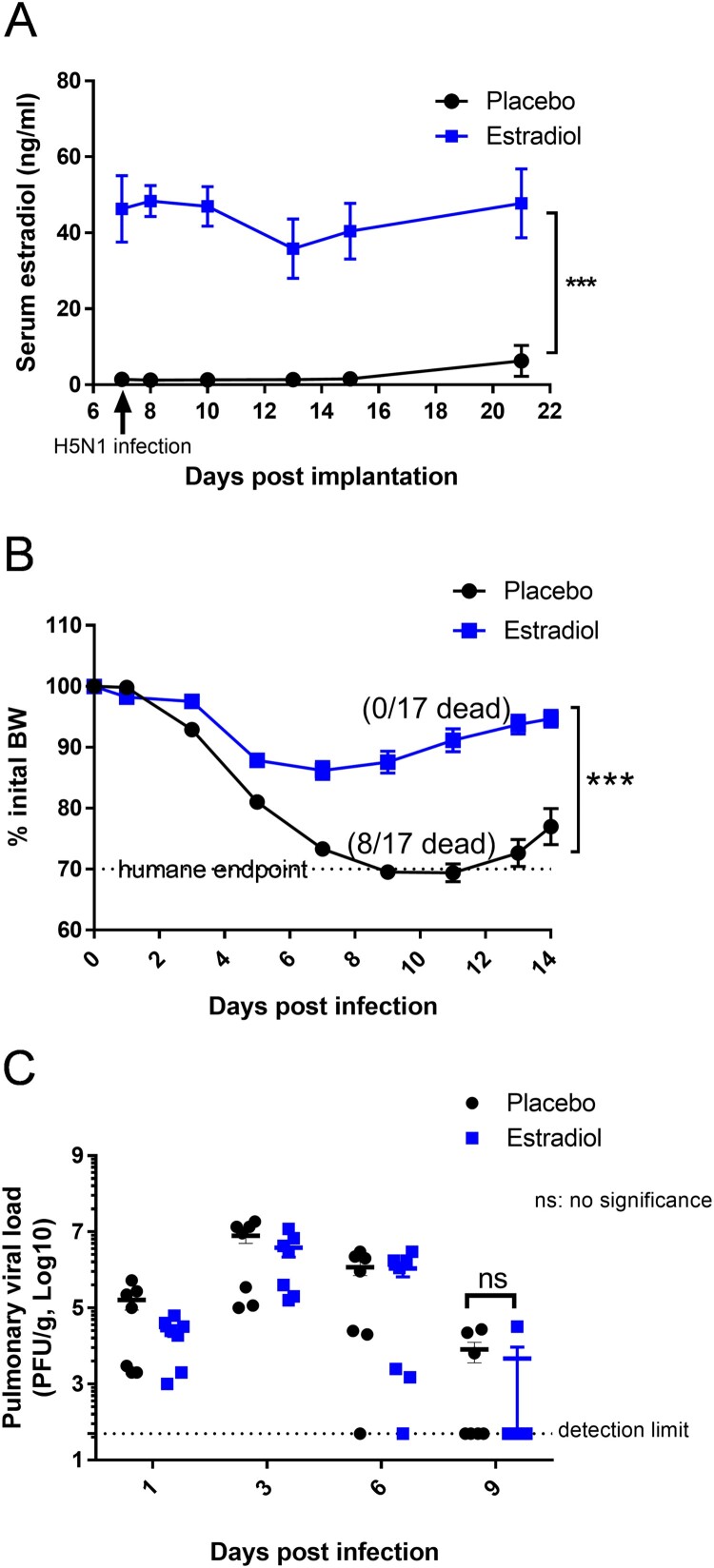
Female mice implanted with estradiol pellets were protected from H5N1 infection. Non-pregnant female Balb/c mice implanted with estradiol or placebo pellets were infected with H5N1 as described above. (A) Serum estradiol levels (*n *= 9–17 mice/group/time point); (B) Body Weight (BW) (*n* = 17 mice/group) and (C) pulmonary viral load (*n* = 7 mice/group/time point). Data are expressed as mean ± SEM. Serum estradiol levels and BW were analyzed by two-way ANOVA. Pulmonary viral titers were analyzed by Mann–Whitney test after log transformation. ***p < 0.001. Data are representative of 2–3 independent experiments with similar results.

To investigate how pregnancy level of estradiol, independent of other pregnancy-associated steroids, affected host immunity against H5N1 infection, non-pregnant female mice were implanted subcutaneously with 35-mg estradiol pellets. Their serum estradiol concentrations were increased above the placebos (Figure 2(A)) and reached levels comparable to those of pregnant mice [22,33]. While H5N1-infected placebo mice exhibited nearly 30% BW loss and 47% mortality, non-pregnant female mice with pregnancy level of estradiol showed transient BW drop and were 100% protected from H5N1 infection (Figure 2(B)). There was no difference in the kinetics of pulmonary virus replication between estradiol-implanted mice and those receiving placebo (Figure 2(C)). These results suggested that estradiol-mediated protection could not be attributed to reduced virus replication in the lungs.

### Pregnancy level of estradiol suppressed pulmonary pro-inflammatory responses to H5N1 infection

**Figure 3.**
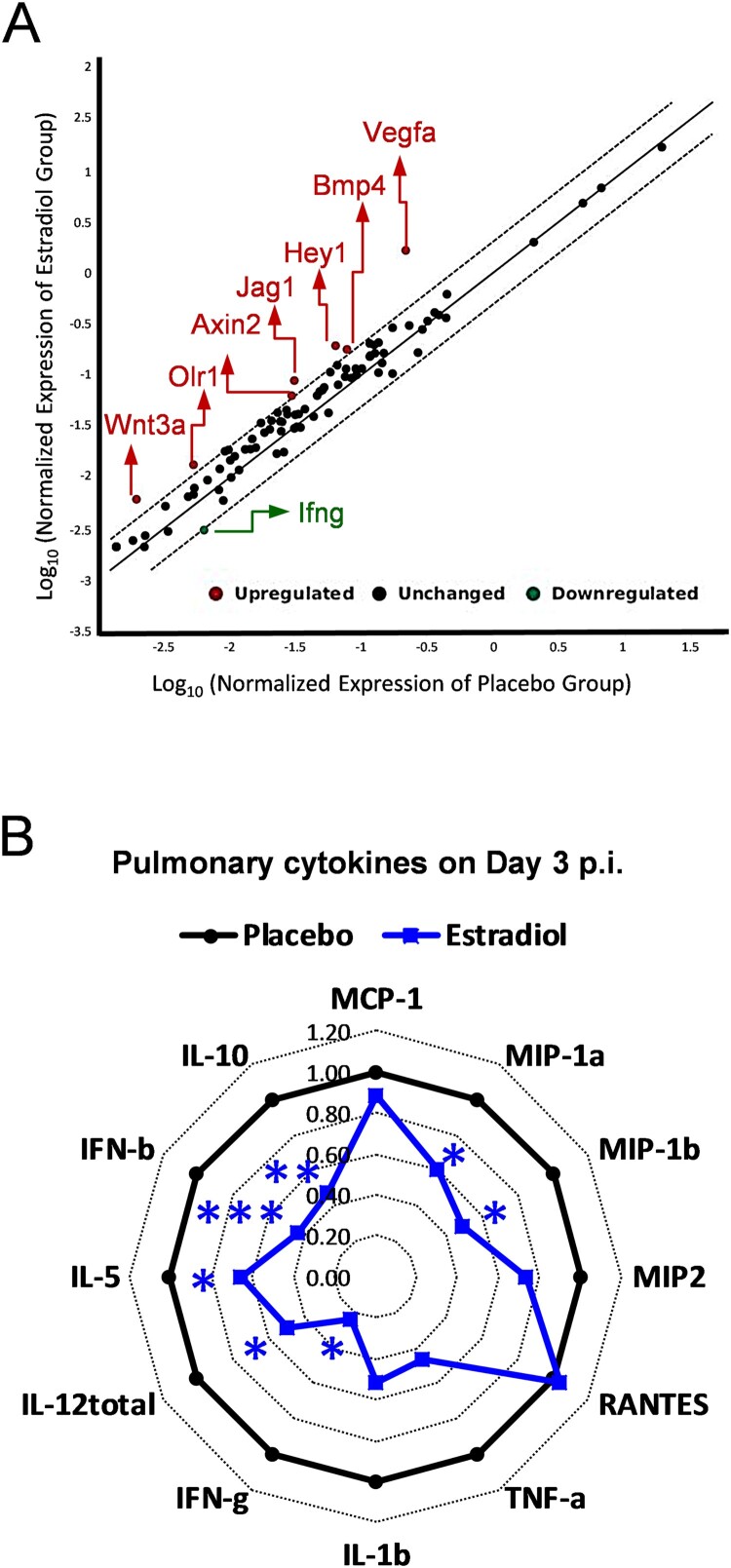
Pregnancy levels of estradiol suppressed pulmonary pro-inflammatory responses to H5N1 infection. (A) Quantitative RT-PCR of pulmonary gene expression of mice implanted with estradiol or placebo pellets on Day 1 post infection (p.i.) with H5N1 virus. The average fold changes of individual genes vs. placebo group are presented in the correlation scatter plot with the dash lines indicating 2-fold change (*n* = 4 mice/group). (B) Radar chart of pulmonary cytokines on Day 3 p.i. (fold inductions vs. placebos, *n *= 7 mice/cytokine/group). *p < 0.05 and *** *p*  <  0.001 compared to placebo by unpaired Student’s *t*-test, respectively. Data are representative of 2–3 independent experiments with similar results.

To determine which signal transduction pathways were activated by estradiol implantation immediately after H5N1 infection, we performed quantitative RT-PCR array on lungs harvested on day 1 p.i. While most genes remained unchanged as compared to the placebo group (within the 2-fold diagonal dashing lines), estradiol-implanted mice showed significant up-regulation of *Vegfa* (Hypoxia signaling), *Hey1* and *Jag1* (Notch signaling), *Bmp4 and Wnt3a (Hedgehog signaling), Olr1* (PPAR signaling), and *Axin2* (WNT signaling) and significant downregulation of *ifng* (*NF-kB* signaling) (Figure 3(A)). Meanwhile, the pulmonary cytokine profiling revealed significantly reduced pro-inflammatory cytokines/chemokines (MIP-1α, MIP-1β, IFN-γ, IL-5, IL-12_total_, IFN-β and IL-10) in estradiol-implanted mice as compared to placebos on day 3 p.i., (*p* < 0.05, Figure 3(B) and Supplementary Figure S3). While the differences in most lung cytokines/chemokines disappeared after this acute phase of infection, estradiol-implanted mice continued to show significant reductions in pulmonary MIP-1α and MIP-1β levels than placebo mice after day 6 p.i. (data not shown). The reduced lung inflammation along with unaltered viral load in estradiol-implanted mice suggested that estradiol-induced anti-inflammatory effects likely contributed to the protection against H5N1 infection.

### Pregnancy level of estradiol reduced antibody responses after H5N1 infection

**Figure 4.**
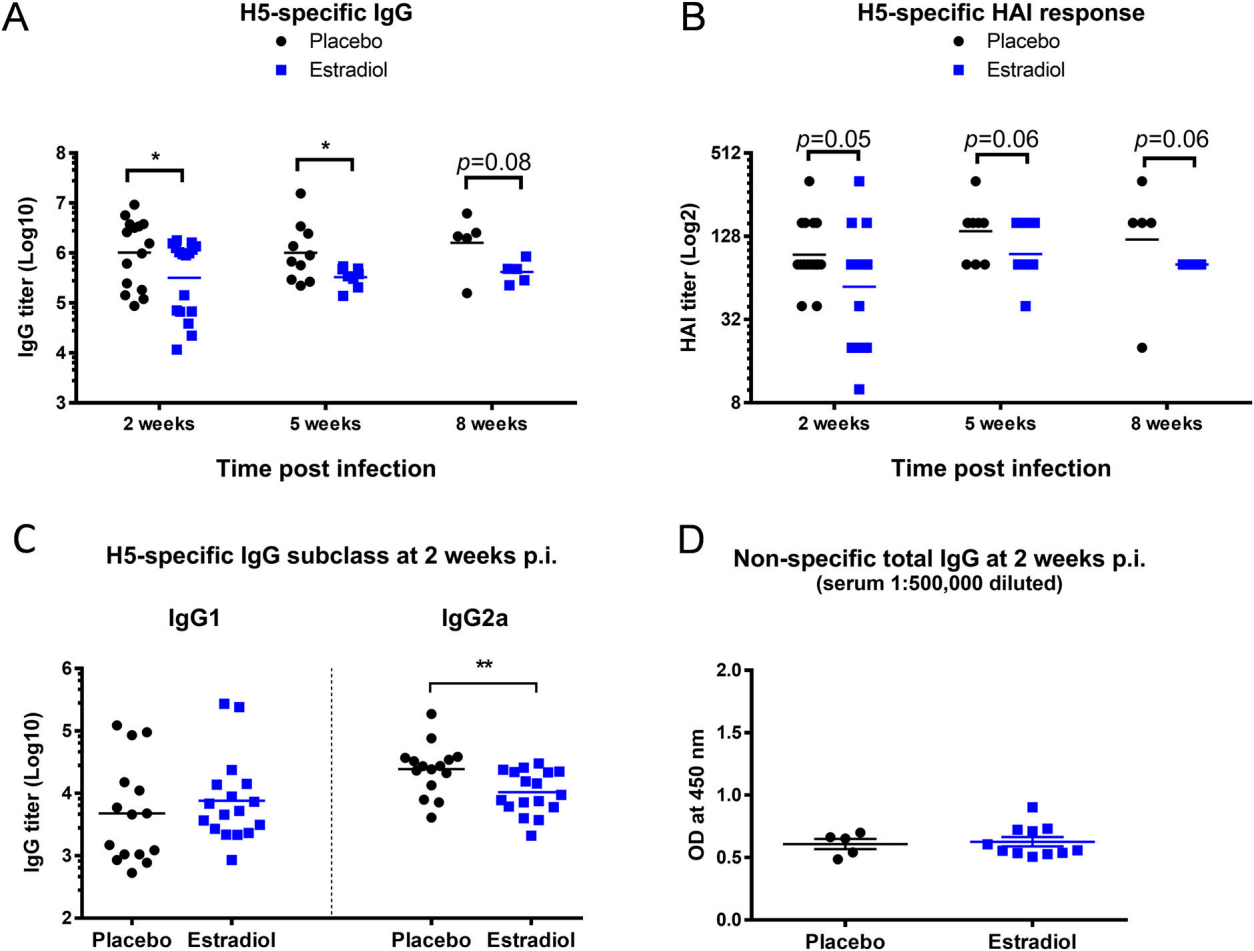
Pregnancy level of estradiol suppressed humoral response to H5N1 infection. Sera of estradiol- and placebo-implanted female mice (*n* = 5–17 mice/group/time point) were collected at different time points of H5N1 post infection (p.i.) for antibody determination. (A) H5-specific IgG titers with geometric mean (lines); (B) H5-specific HAI titers with geometric mean (lines); (C) H5-specific IgG1 and IgG2a titers with geometric mean (lines); (D) non-specific total IgG (OD mean ± SEM). *p < 0.05 and **p < 0.01 by Mann–Whitney test after log transformation. Data are representative of 2–3 independent experiments with similar results.

We next investigated if pregnancy level of estradiol affected virus-specific antibody development after H5N1 infection. Compared to the surviving placebos, estradiol-implanted mice exhibited significantly lower serum IgG titers (*p* < 0.05, Figure 4(A)) and substantially lower H5-specific HAI titers (*p* = 0.05, Figure 4(B)) at 2 weeks p.i. The reduced H5-specific antibody response in estradiol-implanted mice, which was observed as early as 9 days p.i. (Supplementary Figure S4) lasted up to 8 weeks p.i. (Figure 4(A,B)). IgG subclass analysis revealed that estradiol-implanted mice had a slightly higher H5-specific IgG1 but significantly lower IgG2a response (*p* < 0.01) compared to placebo controls (Figure 4(C)). The reduced antibody response in estradiol-implanted mice was H5-specific since these mice and the placebo group had similar levels of non-specific total IgG after H5N1 infection (Figure 4(D)). Additionally, serum estradiol concentrations appeared inversely correlated with H5-specific antibody titers: higher estradiol levels, lower antibody titers (Supplement Figure S5A, S5B & S5C). These results indicate that the pregnancy level of estradiol, despite induction of anti-inflammatory protection against H5N1 infection (Figures 2 and 3(B)), attenuated virus-specific antibody development in implanted mice.

### Pregnancy level of estradiol suppressed H5-specific IgG and IL-4 ELISPOT responses

**Figure 5.**
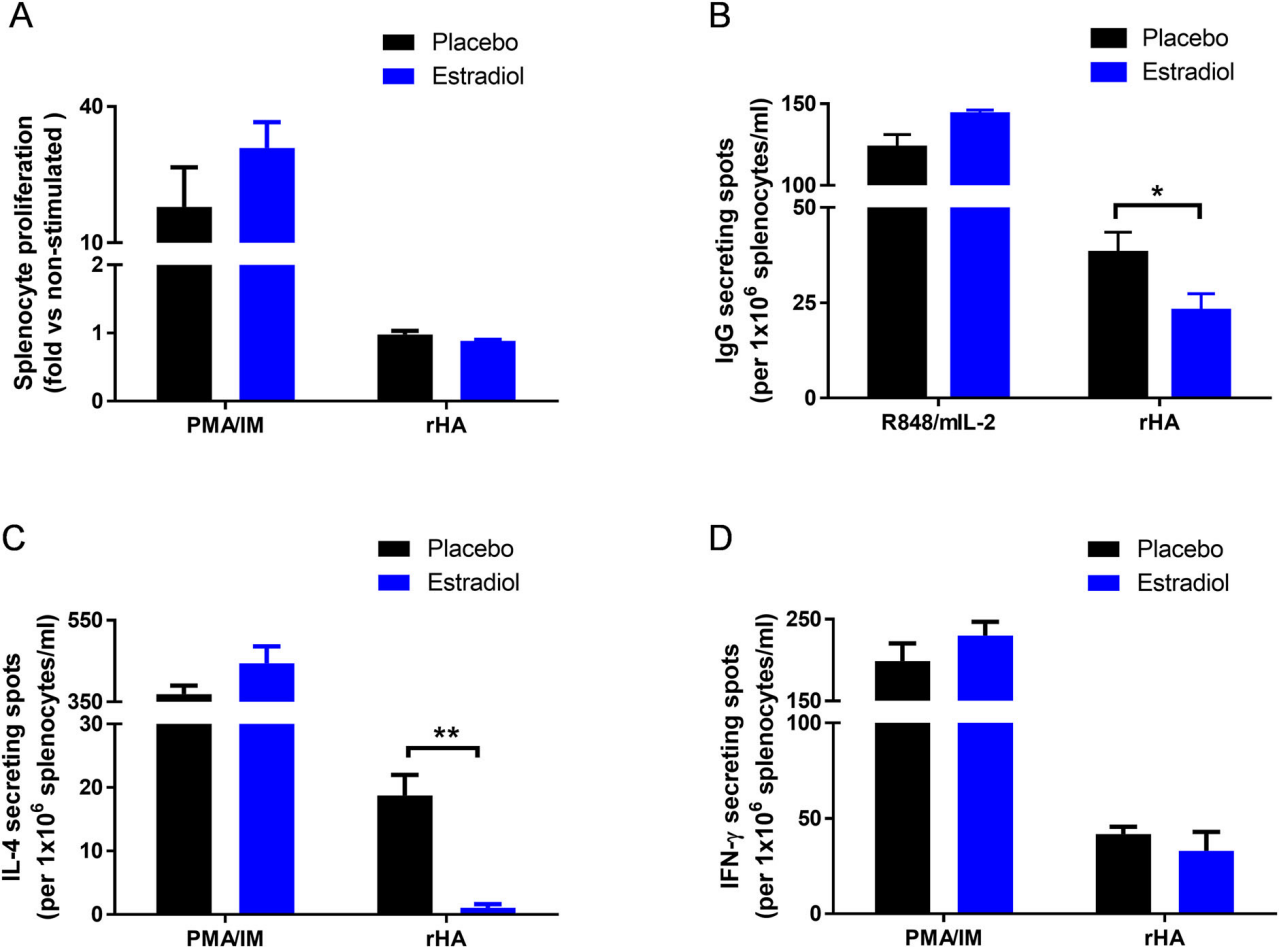
Effects of estradiol on splenocyte proliferation and cytokine ELISPOT responses to H5N1 infection. Estradiol- and placebo-implanted female mice were infected with H5N1 as described above. Splenocytes harvested at 7 weeks post infection were stimulated in vitro with H5 recombinant HA (rHA), PMA/ionomycin (PMA/IM) mixture or R848/mIL-2 mixture. (A) Splenocyte proliferation, and (B) IgG, (C) IL-4 and (D) IFN-γ secreting cells measured by ELISPOT. Data are expressed as mean ± SEM (*n* = 3 mice/group) and are representative of 2–3 repeated experiments with similar results. *p < 0.05 and **p < 0.01 by unpaired Student’s *t*-test.

Cell proliferation and ELISPOT assays were performed to reveal the effects of pregnancy level of estradiol on cellular immunity in response to H5N1 infection. Splenocytes from estradiol-implanted or placebo mice after H5N1 infection showed no difference in cell proliferation toward H5 or PMA/ionomycin (PMA/IM) re-stimulation in vitro (Figure 5(A)). However, estradiol-implanted mice had significantly lower H5-specific IgG-secreting cells than placebo mice (*p* < 0.05) while non-specific IgG response toward R848/mIL-2 stimulation remained unaffected (Figure 5(B)). Estradiol-implanted mice also exhibited significantly reduced frequencies of H5-specific IL-4 (*p* < 0.01, Figure 5(C)) but not IFN-γ secreting cells as compared to placebo mice (Figure 5(D)). In contrast, splenocytes from estradiol-implanted or placebo mice showed no difference in IL-4 or IFN-γ secretion in response to non-specific PMA/IM stimulation (Figure 5(C,D)). These results suggest that pregnancy level of estradiol suppressed H5-responsive IgG-secreting cells and IL-4-secreting cells after H5N1 infection.

### Pregnant women had lower antibody titers than non-pregnant females after seasonal influenza vaccination

**Figure 6.**
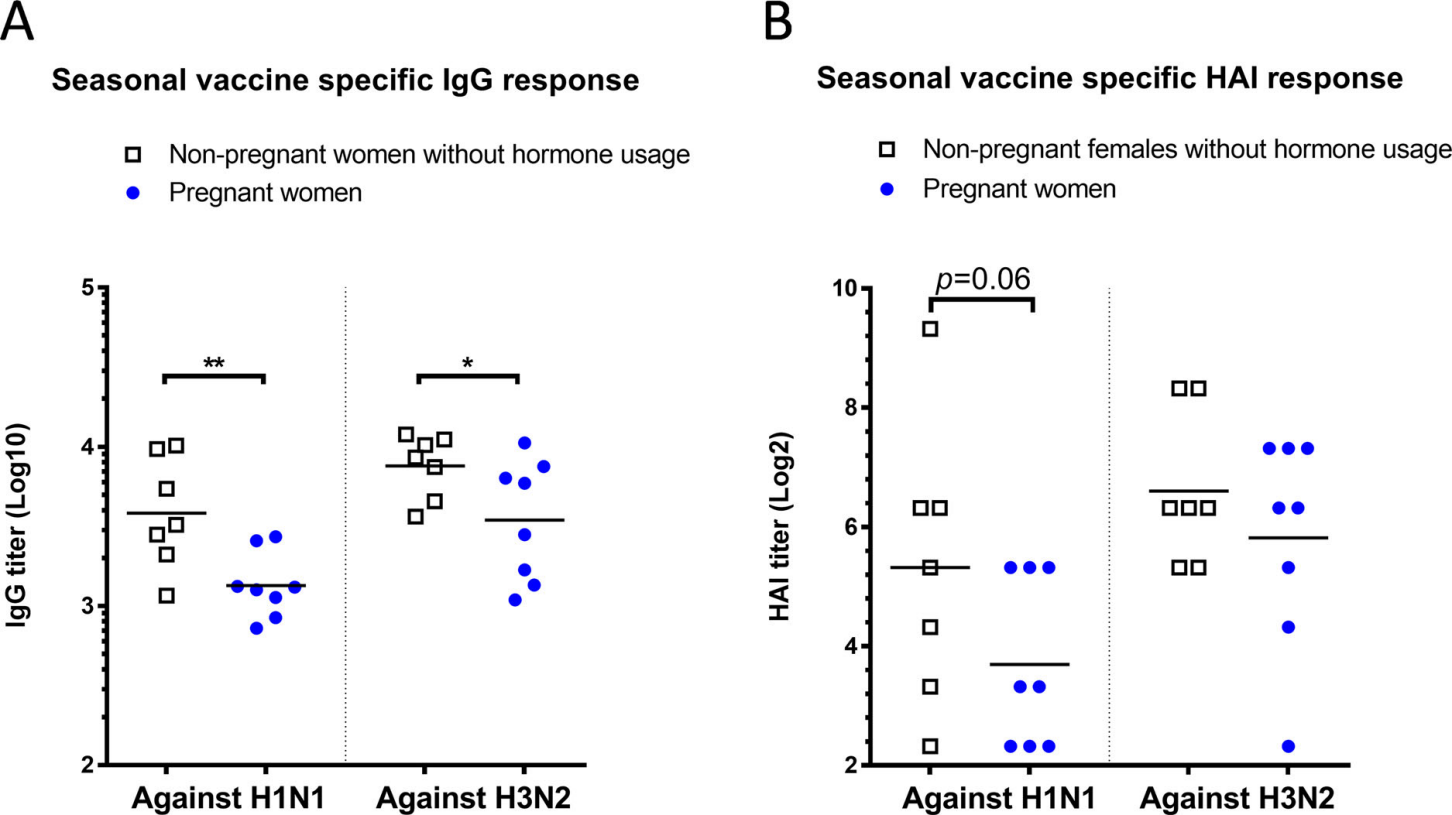
Influenza-specific antibody responses in non-pregnant and pregnant women. Post-seasonal influenza vaccination responses in sera from pregnant women and non-pregnant women without hormone usage were determined, including (A) IgG and (B) HAI titers specific for H1N1 vaccine strain A/Michigan/45/2015 and H3N2 vaccine strain A/Hong Kong/4801/2014. Individual titers (*n *= 7–8 subjects/group) and geometric mean (lines) are shown. * *p*  <  0.05 and ** *p*  <  0.01 by Mann–Whitney test after log transformation.

Since our data revealed that pregnancy level of estradiol suppressed influenza-specific antibody response in non-pregnant female mice with intact ovaries, we asked whether this observation would be relevant to pregnant women receiving influenza vaccines. Compared to age-matched non-pregnant women who did not take birth control medications, pregnant women who were vaccinated during pregnancy developed significantly lower IgG titers toward both H1N1 and H3N2 vaccine strains of the 2017/18 seasonal influenza vaccines (*p* < 0.01 toward A/Michigan/45/2015 H1, and *p* < 0.05 toward A/Hong Kong/4801/2014 H3, Figure 6(A)). Pregnant women also exhibited approximately 70% and 40% lower post-vaccination HAI geometric mean titers (GMTs) than non-pregnant female subjects against H1N1 A/Michigan/45/2015 (GMT: 13 vs. 40) and H3N2 A/Hong Kong/4801/2014 (GMT: 57 vs. 98), respectively (Figure 6(B)). These results, though obtained with small sample size, suggest that pregnant women may likely experience suppressed antibody responses toward influenza vaccination than non-pregnant females without hormone treatments.

## Discussion

Sex-dependent and pregnancy-associated vulnerability to influenza infections has been well documented in humans [2,3,6,34]. Pregnant women are particularly susceptible to avian influenza H5N1 infections and are more likely to develop severe complications [3,8,9,10]. Studies have been conducted in animal models to understand sex-biased differences and pregnancy-associated susceptibility to seasonal influenza infections, mainly on H1N1 viruses [15,22,26,27,35,36]. However, limited experimental evidence is available to understand H5N1-related sex bias and pregnancy-associated susceptibility. In this study, we demonstrated heightened susceptibility of female and pregnant mice to avian influenza H5N1 infections (higher morbidity and higher mortality than male mice). H5N1 infection also induced stronger local or systemic inflammatory reactions in infected female and pregnant mice. Others also reported that female and pregnant mice infected with H1N1 viruses had significantly higher pulmonary secretion of pro-inflammatory cytokines/chemokines than H1N1-infected male and non-pregnant female mice, respectively [15,25,26]. Moreover, elevated pro-inflammatory cytokines have been linked to increased disease severity and death in pandemic H1N1-infected pregnant women [37]. Together, these results suggest that cytokine dysregulation is a heightened risk of influenza infections in both non-pregnant and pregnant women. Similar to a previous report on H1N1-infected female rats [38], our study also indicates that female mice, unlike male mice, appeared to mount Th2-biased proinflammatory reactions (higher pulmonary IL-5) to H5N1 infection. These sex-related differences likely reflect differential modulation of male and female hormones on host immunity, with female sex steroids such as estradiol and progesterone favoring a Th2-biased immunity [11,16,17,39,40,41].

Estradiol at high concentrations has immunosuppressive effects, and elevated estradiol during pregnancy is believed to maintain immune tolerance to support the embryonic implantation and prevent fetus rejection [11]. In this study, we demonstrated that pregnancy level of estradiol, independent of other pregnancy-associated hormones, prevented severe morbidity and improved survival rate in H5N1-infected non-pregnant female mice. This improvement of infection outcome was not due to inhibition of pulmonary virus replication. Instead, it was attributed to estradiol-induced pulmonary reduction of pro-inflammatory cytokines and chemokines. Similar findings have been reported in H1N1-infected animals, in which estradiol reduced influenza-related lung inflammation but had no impact on pulmonary replication of influenza viruses [15,22,23,24,38]. Our data further revealed that the estradiol-mediated control of lung inflammation was apparently regulated via augmented hypoxia and Notch signaling and down-regulated *NF-k*B signaling. Under hypoxia, estradiol can boost *Vegfa*-dependent Notch activation to promote endothelial cell survival, thus playing a role in cardiovascular protection [42]. Estradiol at high levels is also known to suppress the transcription of proinflammatory genes via the *NF-k*B pathway [43]. These results seemed to indicate that the anti-inflammatory properties of estradiol (at high systemic concentrations) may be beneficial in preventing inflammation-associated lung damage during avian influenza H5N1 infections.

In this study, we also observed that estradiol-implanted mice developed significantly lower virus-specific antibody responses than placebos after H5N1 infection, despite both groups having similar pulmonary viral loads during acute infection. This reduced antibody response in mice with pregnancy level of estradiol was antigen-specific. IgG subclass analysis also revealed that estradiol-implanted mice had significantly lower IgG2a (Th1 in mouse) after H5N1 infection, indicating a Th2-biased shift in virus-specific humoral immunity. Reduced IgG2 (Th1 in human) responses have also been observed in pandemic H1N1-infected pregnant women [44,45]. Moreover, the reduced H5-specific antibodies along with reduced H5-specific IgG-secreting cells indicate an impaired B cell response in estradiol-implanted mice after H5N1 infection. A significant reduction in IgG-specific B cells was also observed in pandemic H1N1-infected pregnant ferrets [27]. It has been reported that pregnancy levels of estradiol can reduce the production of progenitor B cells in murine bone marrow and adversely affect the differentiation and survival of progenitor B cells in the spleen [46,47]. This may cause a delay in eliciting antigen-specific B cells resulting in attenuated antibody response after pathogen exposure.

Additionally, pregnancy levels of estradiol have been shown to suppress T cell responses and decrease antigen-specific IL-2 and IL-4 secretion by Th2 lymphocytes [11,22,48]. In this study, we observed that splenocytes from estradiol-implanted mice had significantly lower frequencies of IL-4-secreting cells, but unaltered IFN-γ-secreting cells than placebos after H5 HA re-stimulation, indicating a reduced virus-specific Th2 cell response. Similar phenomena have also been observed in pandemic H1N1-infected pregnant women who had diminished CD4+ Th2 cells but elevated IFN-γ responses as compared to infected non-pregnant women [44]. The peripheral blood mononuclear cells isolated from H1N1-infected pregnant ferrets also secreted significantly lower IL-4 but notably higher IL-12p40 than those of H1N1-infected non-pregnant ferrets [27]. The shift from Th1- to Th2-type cytokine production during gestation is critical to maintain normal pregnancy and avert fetal rejection [21]. However, influenza infections during pregnancy may break this balance resulting in aberrant Th1/Th2 ratio which could potentially endanger pregnancy and cause poor birth outcomes.

We showed herein that pregnancy level of estradiol alone could reduce virus-specific humoral responses after H5N1 infection. This scenario could be compounded in real pregnancy when other steroids are present. For example, estriol – another major estrogen produced during pregnancy has been reported to negatively impact progenitor B cell development and reduce antibody response after infection [46,49]. This estrogen-induced suppression of humoral immunity is further complicated by progesterone, which can sensitize B-cell precursors to the negative regulation of estradiol at even lower concentrations, despite the fact that progesterone alone does not affect B-cell lineage development [46]. As a result, virus-specific antibody can be dampened in pregnant women as pregnancy progresses to full term. In this study, we observed that pregnant women developed significantly lower antigen-specific antibody titers after 2017/18 seasonal vaccination than non-pregnant women without hormone treatment. Schlaudecker et al. have also reported that pregnant women have reduced antibody responses following seasonal influenza vaccinations, which worsens as pregnancy progresses [50,51]. Impaired antibody responses against influenza viruses during pregnancy not only jeopardize pregnant women’s health but also confer insufficient protection for fetuses and newborns. As shown in this study and the study by Littauer et al. [26], pups born from influenza-infected dams had significantly retarded growth and increased mortality.

In summary, our study demonstrated that pregnancy level of estradiol has divergent effects on host immune response against H5N1 infection – its anti-inflammatory properties can ameliorate infection-associated lung inflammation in mice, but it also can hinder virus-specific antibody development after infection. Since newborns obtain passive immunity via breastfeeding, our results emphasize the necessity of influenza vaccination during pregnancy and provide impetus to formulate strategies to increase vaccine coverage among pregnant women.

## Supplementary Material

Supplemental Material

## Data Availability

Quantitative RT–PCR array data are available on NCBI Gene Expression Omnibus (GEO) under the accession number GSE128447.
