# Supplementary material for: Pregnancy level of estradiol attenuated virus-specific humoral immune response in H5N1-infected female mice despite inducing anti-inflammatory protection
Source: Emerg Microbes Infect. 2019 Jul 31;8(1):1146–56. doi: 10.1080/22221751.2019.1648184 (PMC6711175; doi:10.1080/22221751.2019.1648184)

**Pregnancy Level of Estradiol Attenuated Virus-specific Humoral Immune Response in H5N1-infected Female Mice Despite Inducing Anti-inflammatory Protection**

**Courtney L. Finch^a^, Anding Zhang^a^, Martina Kosikova^a^, Toshiaki Kawano^a^, Marcela F. Pasetti^b^, Zhiping Ye^a^, Jill R. Ascher^c^, Hang Xie^a*^**

^a^Division of Viral Products, Center for Biologics Evaluation and Research, United States Food and Drug Administration, Silver Spring, Maryland, USA

^b^Department of Pediatrics, Center for Vaccine Development and Global Health, University of Maryland School of Medicine, Baltimore, Maryland, USA

^c^Division of Veterinary Services, Center for Biologics Evaluation and Research, United States Food and Drug Administration, Silver Spring, Maryland, USA

**^*^**Address correspondence to Hang Xie ([Hang.Xie@fda.hhs.gov](mailto:Hang.Xie@fda.hhs.gov)).

**Supplementary Materials**

**H5N1 Infection in Mice**

All mice used in this study including age-matched non-pregnant female and male adult Balb/c mice, or timed-pregnant Balb/c mice (after 13 days of gestation) were purchased from Charles River laboratories (Frederick, MD) and were used for H5N1 infection at approximately 16-week old. Mouse pregnancy was determined based on vaginal plugs observed in female mice. The date the copulatory plugs observed in female mice after mating was considered day one of gestation. Mouse pregnancy was confirmed by palpation after 13 days of gestation (<https://www.criver.com/sites/default/files/resources/PregnantAnimalGuaranteePolicy.pdf>).

All mice were housed in Biosafety Level (BSL)-2 facility at 5/microisolater cage for female or male mice, and 4/microisolater cage for pregnant mice until infection. Food and water were available *ad libitum*. Newly arrived mice including pregnant mice were given 4-5 days to acclimate to the new environment before any experimental procedures were performed.

The human H5N1 A/Vietnam/1203/2004 (VN/1203) vaccine strain bearing a monobasic cleavage site in HA remains fully pathogenic in mice [1]. Its replication in mouse lungs reaches the peak on day 3 post infection (p.i.) and returns to the baseline by day 9 p.i. [1]. VN/1203 had been titrated in male Balb/c mice. A sublethal dose of VN/1203 at 500 PFU/50µl/mouse that caused 0% mortality in male Balb/c mice while had nearly half of infected female mice succumbed to death was determined. Age-matched (approximately 16-week old) non-pregnant female and male adult Balb/c mice were infected intranasally with VN/1203 at 500 PFU/50µl/mouse under isoflurane anesthesia. Infected mice were under close observation for disease development. Body weight (BW) was monitored daily for up to 14 days post infection (p.i.). Mice reaching humane endpoints (e.g. 30% BW loss, rapid/labored breathing or inability to move or respond to human touch) were immediately euthanized. Lungs were harvested on day 3 p.i. (the peak of pulmonary virus replication) for cytokine determination.

Pregnant mice were infected on 18 days of gestation with VN/1203 (500 PFU/50µl/mouse) as described above and were individually housed in microisolater cages thereafter to allow more space for litters delivered later. Infected dams were monitored BW daily for up to 14 days p.i. BW and mortality of pups born to H5N1-infected dams were also monitored and recorded daily. All pups were humanely euthanized on 7 days after the delivery. To minimize the stress caused to dams, sera were collected on 10 days p.i. (7 days after the delivery) for systemic cytokine determination beyond viral clearance.

One week before virus infection, non-pregnant female mice were implanted under isoflurane anesthesia with 21-day slow release 17-β-estradiol (10 or 35 mg/pellet/mouse) or similar-sized placebo (Innovative Research of America, Sarasota, FL). The pellets were inserted below the loose skin between the shoulder blades via small incision and secured with surgical wound clips. Implanted mice were given 7 days to recover from the surgery before H5N1 infection as described above. Sera were collected via tail bleeding to confirm estradiol elevation. Lungs were collected on day 1, 3, 6 and 9 p.i. for viral loads. All animal experiments were repeated at least 2-3 times at ≥ 5 mice/group. The animal study protocol under which all procedures were performed is approved by the FDA White Oak Animal Program Animal Care and Use Committee in an AAALAC International-accredited animal facility under Animal Biosafety Level (ABSL)-2 plus conditions.

**REFERENCES**

[1] Xie H, Liu TM, Lu X, et al. A live attenuated H1N1 M1 mutant provides broad cross-protection against influenza A viruses, including highly pathogenic A/Vietnam/1203/2004, in mice. J Infect Dis. 2009;200(12):1874-1883.

**Supplement Figures (next pages)**

**Supplement Figure S1.** **Pulmonary cytokine concentrations in male and female mice following H5N1 infection.** Age-matched male and female Balb/c mice were infected with H5N1 virus as described above. Pulmonary cytokine concentrations on day 3 post infection (p.i.) were expressed as mean ± SEM (n=6 mice/cytokine/group). (A) TNF-α, (B) IL-1β, (C) IL-4, (D) IL-5, (E) IL-12total, (F) IFN-γ, (G) mKC and (H) IL-10. **p* < 0.05 by unpaired Student’s *t*-test.


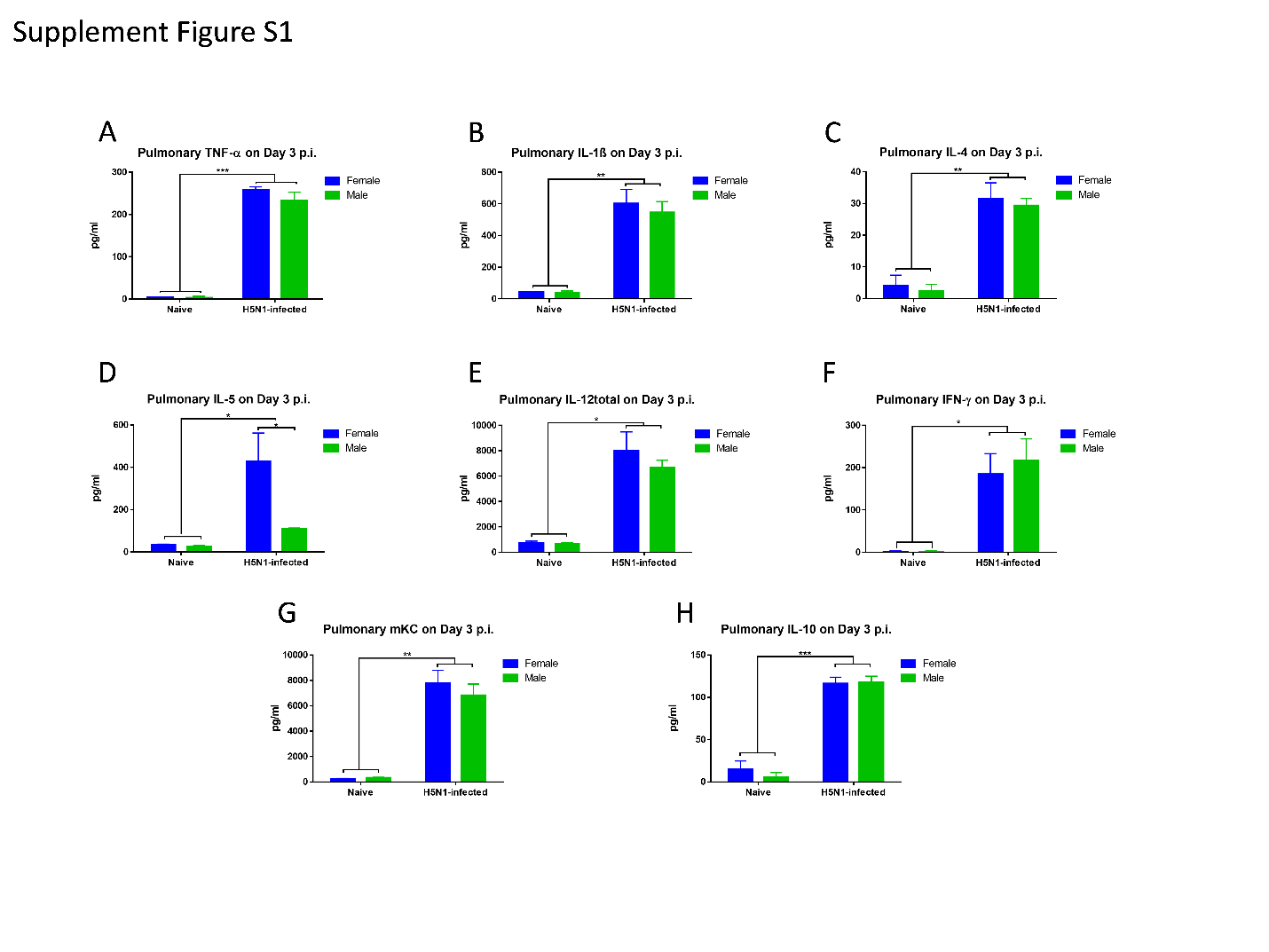


**Supplement Figure S2.** **Serum cytokine concentrations in pregnant and non-pregnant female mice following H5N1 infection.** Pregnant (gestational day 18) Balb/c mice and age-matched non-pregnant female mice were infected with H5N1 virus as described above. Serum cytokine concentrations on day 10 post infection (p.i.) are expressed as mean ± SEM (n=5-8 mice/group). (A) TNF-α, (B) IL-5, (C) IL-4, (D) IL-1β, (E) IL-12total, (F) IFN-γ, (G) mKC and (F) IL-10. **p* < 0.05 by unpaired Student’s *t*-test.


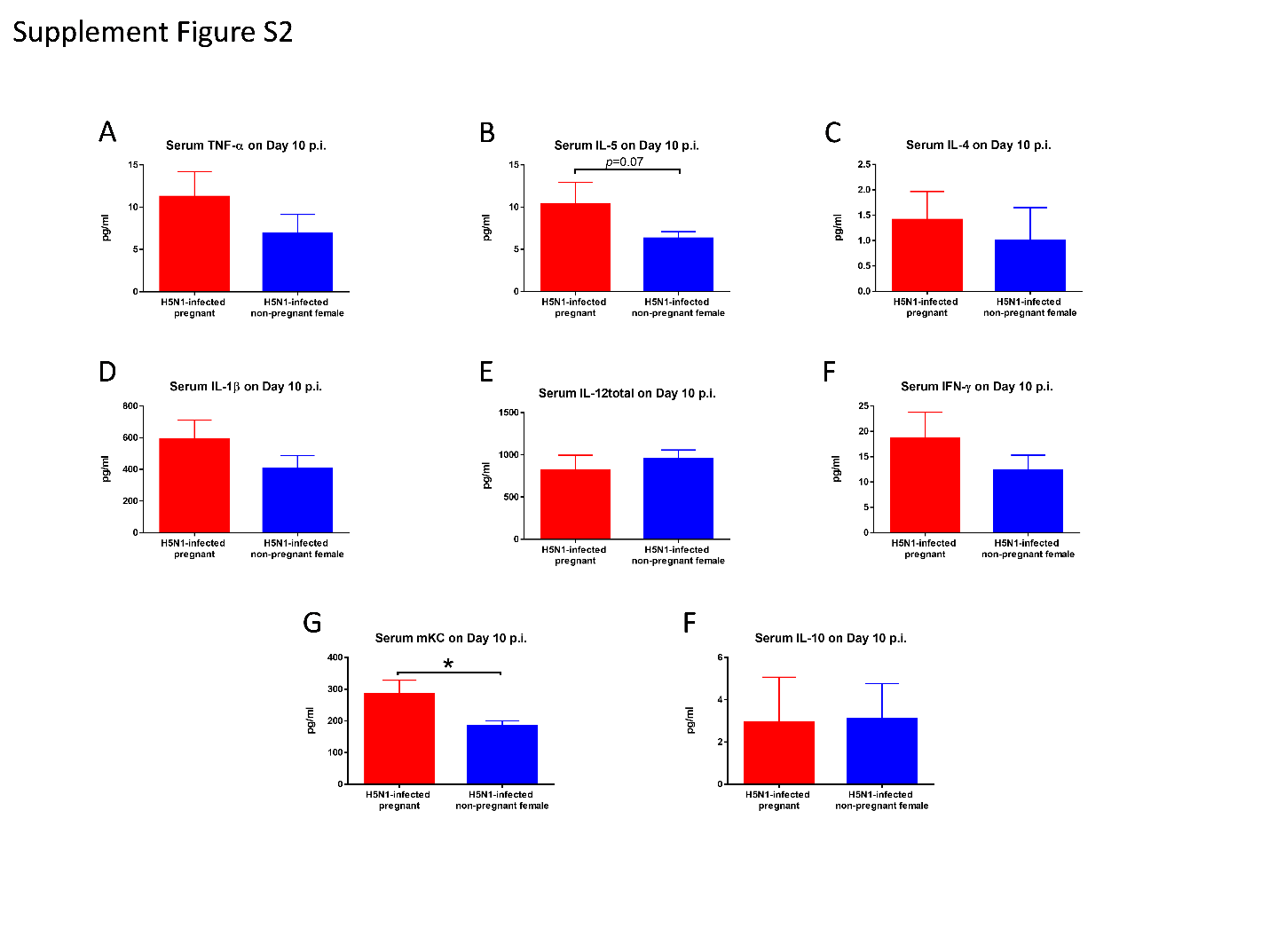


**Supplement Figure S3.** **Pulmonary cytokine concentrations in placebo- and estradiol-implanted mice following H5N1 infection.** Non-pregnant female Balb/c mice implanted with estradiol (35 mg/mouse) or placebo pellets were infected with H5N1 virus as described above. Pulmonary cytokine concentrations on day 3 post infection (p.i.) were expressed as mean ± SEM (n=7 mice/group). (A) MCP-1, (B) MIP-1α, (C) MIP-1β, (D) MIP-2, (E) RANTES, (F) TNF-α, (G) IL-1β, (H) IFN-γ, (I) IL-12total, (J) IL-5, (K) IFN-β and (L) IL-10. * *p* < 0.05, ***p* < 0.01 and ****p* < 0.001 by unpaired Student’s *t*-test. **
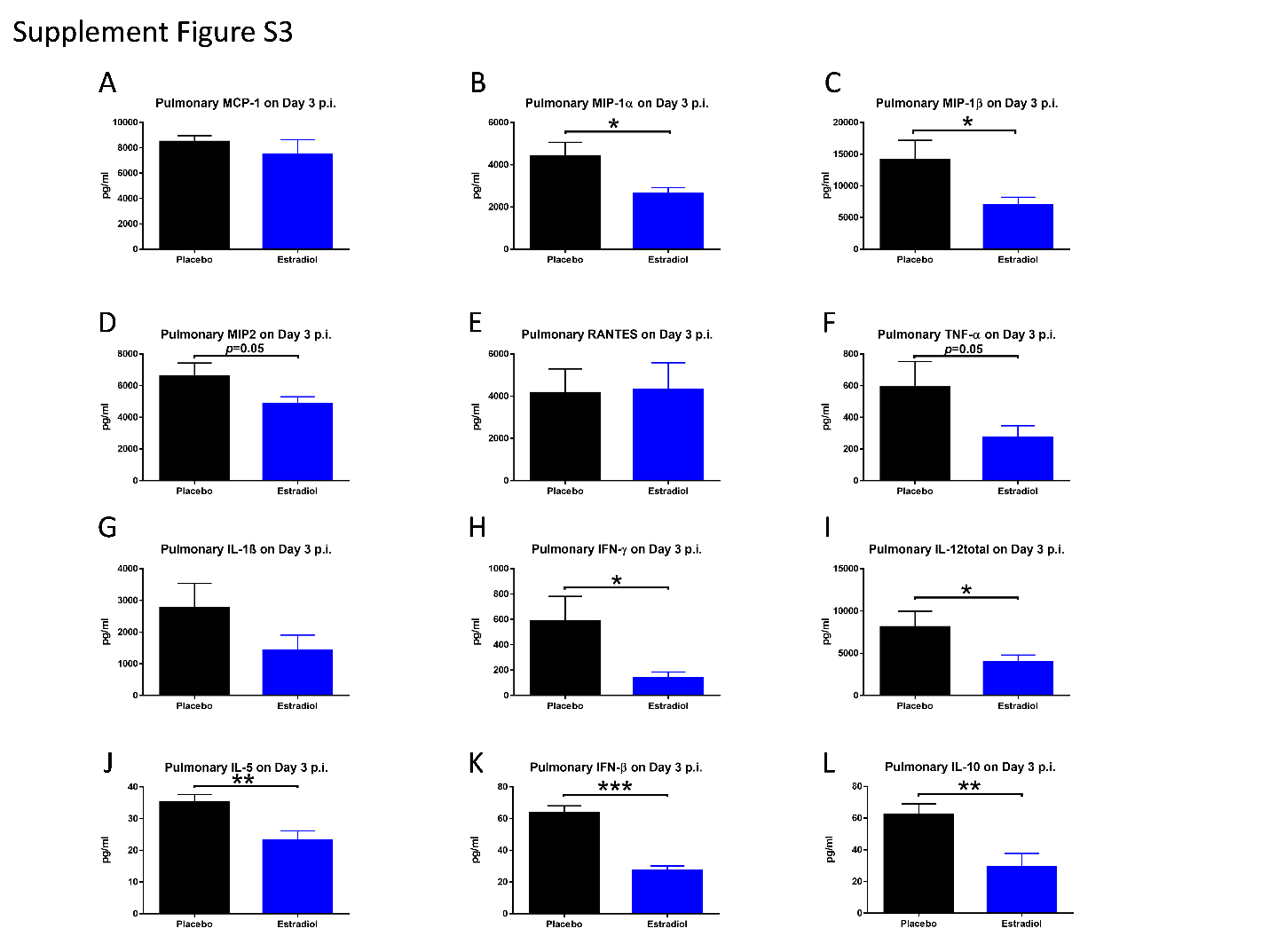
**

**Supplement Figure S4.** **H5-specific IgG titers in placebo- and estradiol-implanted mice on 9 days post infection of H5N1 virus.** Non-pregnant female Balb/c mice implanted with estradiol (35 mg/mouse) or placebo pellets were infected with of H5N1 virus. Sera were sampled on day 9 post infection (p.i.) for H5-specific IgG titers (n=6-7 mice/group) with lines indicating geometric means. *p* value was determined by Mann-Whitney test after log transformation.


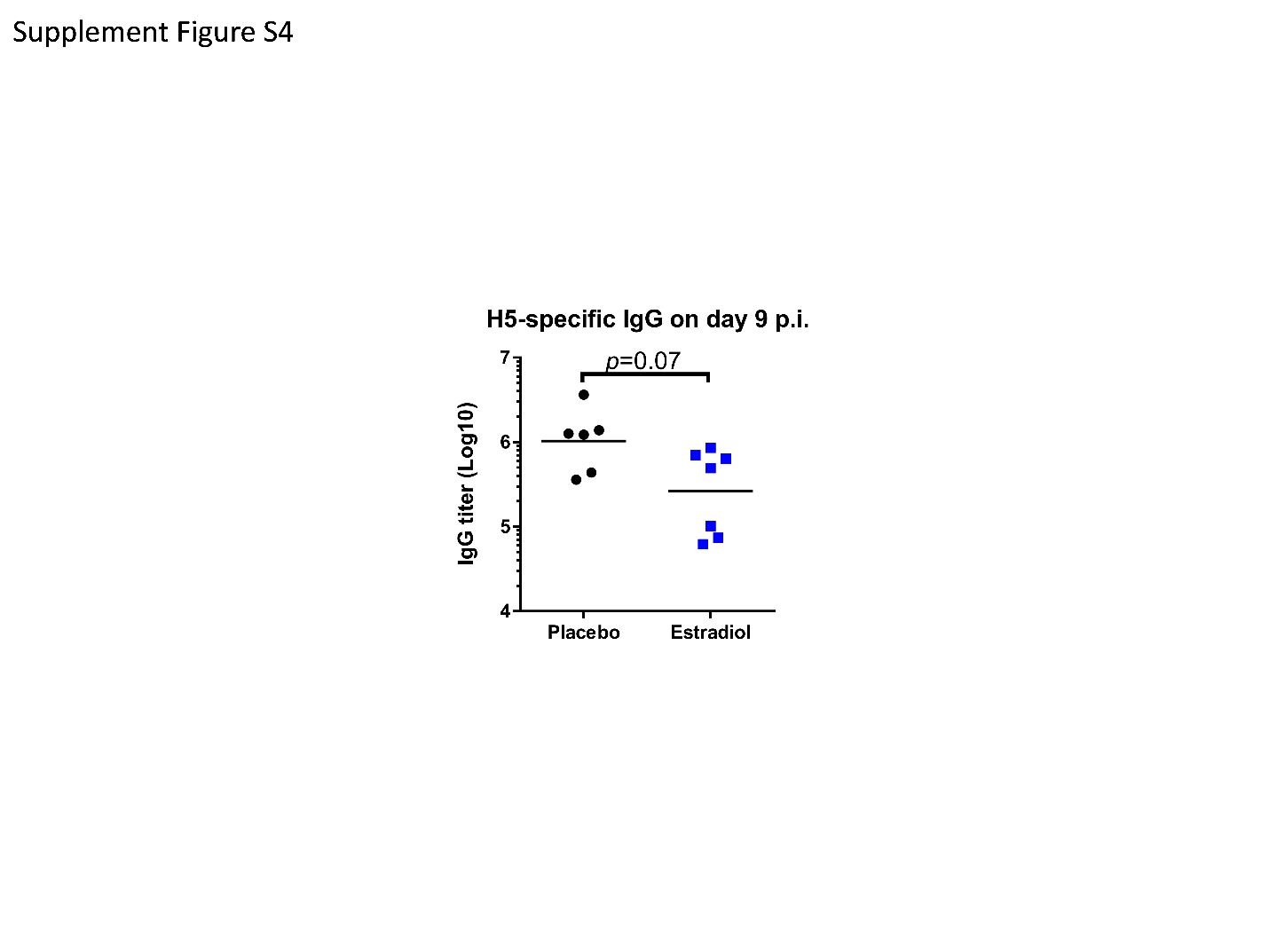


**Supplement Figure S5.** **Serum estradiol concentrations inversely correlated with reduced antibody titers in H5N1-infected mice.** (A) Serum estradiol concentrations in non-pregnant female B/c mice implanted with estradiol (10 mg or 35 mg/pellet) or placebo pellets (n=8 mice/group). (B) H5-specific IgG titers at 2 weeks post infection (p.i.) (lines indicating geometric means). (C) Correlation between H5-specific IgG titers and serum estradiol concentratios by linear regression. * *p* < 0.05 and ** *p* < 0.001 by ANOVA, respectively.


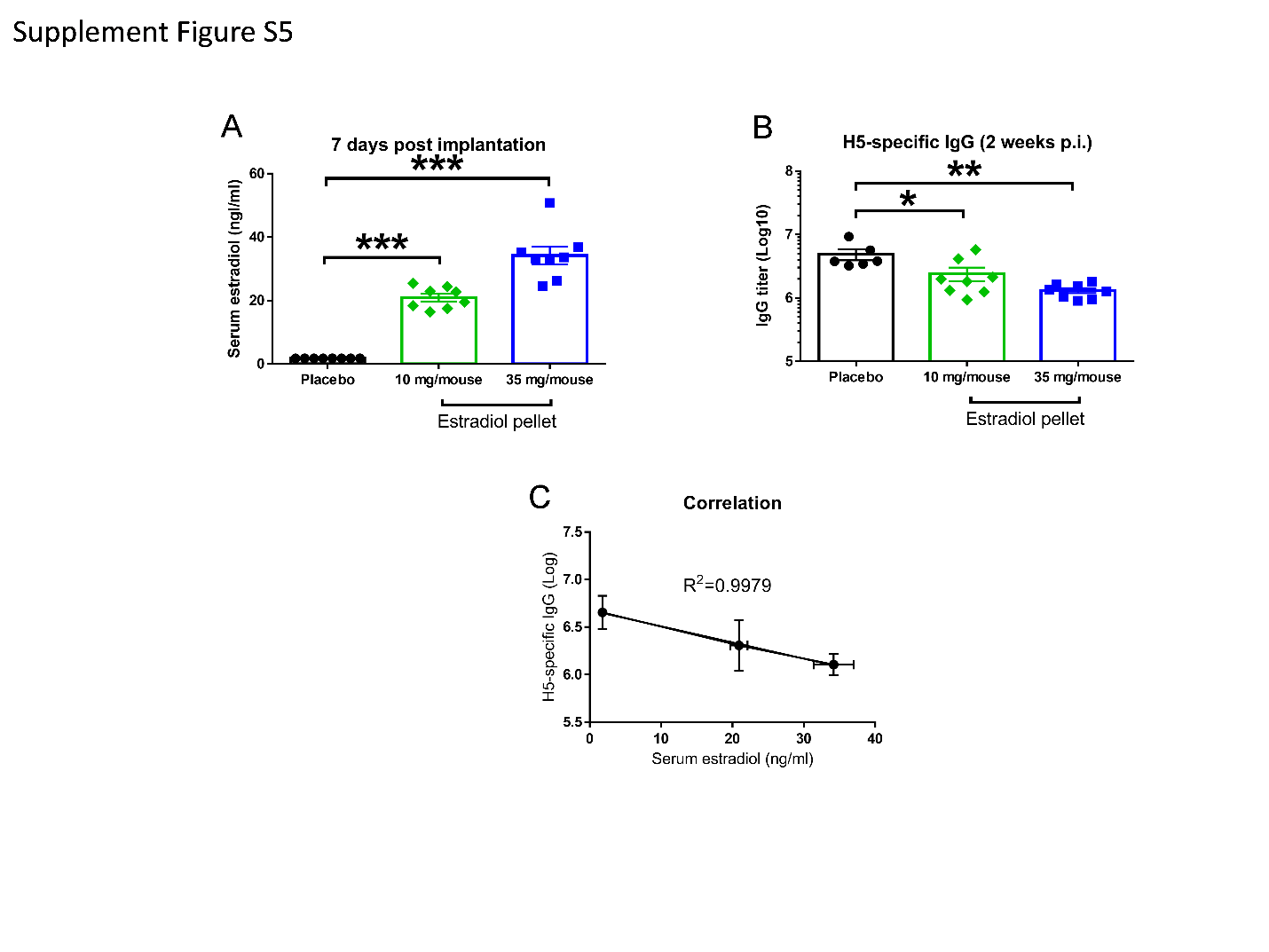

Supplement: Supplemental Material [file TEMI_A_1648184_SM8563.docx]
